# Adipose‐Derived Mesenchymal Stem Cell Secretome for Post‐Steroid Hypopigmentation and Skin Atrophy: A Case Report

**DOI:** 10.1155/crdm/8615178

**Published:** 2026-01-28

**Authors:** Shannaz Nadia Yusharyahya, Valdi Ven Japranata, Endi Novianto

**Affiliations:** ^1^ Department of Dermatology and Venereology, Dr. Cipto Mangunkusumo Hospital, Jakarta, Indonesia; ^2^ Faculty of Medicine, University of Indonesia, Jakarta, Indonesia, ui.ac.id

**Keywords:** adipose-derived mesenchymal stem cells, case report, corticosteroids, postinflammatory hypopigmentation, skin atrophy

## Abstract

Skin hypopigmentation and atrophy are the most commonly reported local adverse effects following corticosteroid application. While these changes are typically reversible, the recovery may be delayed or incomplete in older adults due to diminished physiological reserves and age‐related reductions in melanocyte function and dermal regenerative capacity. Adipose‐derived mesenchymal stem cells (ADMSCs) secretome may offer substantial benefits in reversing the alterations. In this report, we present the clinical evaluation of an elderly patient with steroid‐induced cutaneous manifestations receiving ADMSCs secretome administered with intradermal injection and microneedling. Given that corticosteroid‐induced atrophy and hypopigmentation may improve spontaneously, this case does not establish treatment efficacy; rather, it describes a clinical observation. The potential mechanisms by which ADMSCs secretome may facilitate improvement are also discussed.

## 1. Introduction

Intralesional corticosteroid injection is one of the standard procedures in clinical dermatology practice. While the approach has exhibited usefulness in treating numerous inflammatory and degenerative conditions, postinflammatory hypopigmentation and skin atrophy have been long known as two common local adverse effects, particularly in the skin of colored populations [1]. Through the inhibition of prostaglandin, high doses or prolonged exposure to corticosteroids are postulated to interfere with melanin production of melanocytes and its distribution to adjacent cells, leading to reduced pigmentation [2]. Sometimes, patients may develop hypopigmented linear streaks projected from the injection site, presumably because of corticosteroids’ venous or lymphatic spread [1, 2]. Moreover, their antiproliferative effect on skin cells and suppression of matrix protein synthesis result in decreased skin thickness [1, 3].

These manifestations are usually reversible in several months upon discontinuing corticosteroids; however, they are more profound in the elderly. As individuals age, they will inevitably experience diminished physiological reserves essential to returning their health imbalances to the initial state [4]. This includes their skin’s ability to repair damage caused by internal or external insults and regenerate wounds. On the other hand, regenerative medicine, by applying stem cell products, offers a promising avenue to improve skin changes in older adults, given their self‐renewal and differentiation capacities [5]. Under an optimal environment, adipose‐derived mesenchymal stem cells (ADMSCs) release immunomodulatory cytokines, growth factors, and matrix proteins, known collectively as the secretome. These components regulate biological processes for skin rejuvenation, namely, fibroblast proliferation and collagen synthesis [6], although robust evidence for ADMSCs secretome in alleviating postinflammatory hypopigmentation and skin atrophy is currently lacking. To address this concern, here, we describe the case of an elderly woman with steroid‐induced cutaneous alterations who received ADMSCs secretome. This report aims to document a clinical observation rather than to prove efficacy and to explore potential biological mechanisms that may merit evaluation in controlled studies.

## 2. Case Presentation

A 65‐year‐old female came to our dermatology clinic seeking an esthetic solution for her right knee’s dyspigmented and atrophic lesion. Six months earlier, she went to an orthopedic clinic with a chief complaint of tendon pain in the affected area for the last month, especially when walking and climbing stairs. She had an ideal body weight and practiced running and swimming for physical routines, but she had a history of knee osteoarthritis and flat feet. On physical examination, there was tenderness upon palpation in the anterolateral aspect. Diagnosed with pes anserine bursitis, she underwent treatment with an intrabursal corticosteroid injection (5 mL of triamcinolone acetonide with a concentration of 10 mg/mL), causing hematoma formation due to blood extravasation (Figure 1(a)). She was also prescribed a nonsteroidal anti‐inflammatory drug (NSAID) as a painkiller. One month following the injection, the pain was significantly relieved, but the lesion transformed into a hypopigmented macule with central atrophy and peripheral hyperpigmentation (Figure 1(b)). No apparent improvement was observed without intervention until the presentation to our clinic. Previously, she had already had three intrabursal corticosteroid injections on the same side in the past 2 years with 6‐month intervals because of the same diagnosis, with no side effects.

**Figure FIGURE 1:**
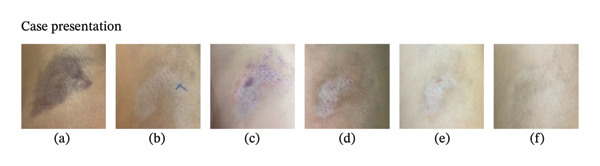
(a) Hematoma on right knee directly after intrabursal injection with triamcinolone acetonide. (b) Hypopigmented macule with central atrophy and peripheral hyperpigmentation after 1 month of corticosteroid injection. (c) Hematoma formation immediately succeeding intradermal ADMSCs secretome administration (Week 0). (d) Pinpoint bleeding after microneedling treatment (Week 2). (e) The lesion following topical ADMSCs secretome application (Week 6). (f) Improvements in hypopigmentation and atrophy (Week 12).

On the first appointment (Week 0), we gave her a 1 mL intradermal injection of allogenic ADMSCs secretome (Figure 1(c)). Two weeks later (Week 2), microneedling with a depth of 150 μm was performed on the lesion until pinpoint bleeding occurred (Figure 1(d)), followed by topical application of 0.25 mL ADMSCs secretome. This procedure was repeated three additional times at 2‐week intervals (Weeks 4, 6, and 8) (Figure 1(e)). No further interventions were performed between Weeks 8 and 12. At Week 12, the patient reported satisfaction as the hypopigmentation had begun to fade and the atrophied skin had risen closer to the normal skin level (Figure 1(f)).

## 3. Discussion

This report presented a clinical case of an elderly woman with recurrent pes anserine bursitis, a local inflammation affecting the bursa between the tibia and the attached three hamstring muscle tendons. The patient’s symptoms were consistent with the diagnosis: pain in the anteromedial aspect of the knee exacerbated by physical activity and palpation on the site. Intralesional corticosteroid injection with triamcinolone acetonide in this patient is a commonly used treatment for pes anserine bursitis and other inflammatory conditions due to its potent anti‐inflammatory effects [7]. Despite the expected pain relief within 1 month of corticosteroid injection in this patient, a hyperpigmented macule developed at the peripheral area of the lesion, and the central portion displayed hypopigmentation and atrophy. The peripheral hyperpigmentation may be attributable to increasing melanin production and distribution secondary to the inflammation [8]. As for the central area alterations, they may result from local inhibitory effect of the injected corticosteroid, including suppression of melanocyte activity through tyrosinase inhibition and reduced melanocyte survival and migration, as well as impaired dermal matrix synthesis due to decreased fibroblast proliferation and collagen synthesis [2, 3]. According to the manufacturer’s information, the triamcinolone acetonide injection used in our patient (Flamicort) is a long‐acting preparation that lasts for 3 weeks. However, her lesion did not improve after 6 months from the cessation of corticosteroid injection. A previous case series has delineated comparable presentations in 24 Indian patients aged 4–45 years, which were regressed spontaneously or after treatment with tacrolimus or platelet‐rich plasma (PRP) within four weeks [9]. We predicted that the elderly status of our subject might interfere with the healing process, as older adults have aberrant melanocyte dispersion in their skin and a relatively lower level of keratinocyte stem cells necessary for skin regeneration [10].

Adding growth factors to revitalize the remaining stem cell reservoir may compensate for our patient’s restricted number of keratinocytes to restore skin conditions. From this idea, we administered an intradermal injection of allogenic ADMSCs secretome, obtained from liposuction specimens of healthy donors undergoing abdominal plastic surgery. The secretome was processed at the Stem Cell Medical Technology Integrated Service Installation, Dr. Cipto Mangunkusumo Hospital. This facility is a government‐accredited stem cell processing center in Jakarta, Indonesia, operating under Good Manufacturing Practice (GMP) standards, and is authorized to produce clinical‐grade mesenchymal stem cell products. Quality analyses were conducted to confirm that the final product was free from aerobic and anaerobic bacteria, endotoxins, fungi, and *Mycoplasma*. Enzyme‐linked immunosorbent assay (ELISA) and Luminex multiplex assay were used to characterize the secretome, revealing the presence of epidermal growth factor (EGF), fibroblast growth factor 4 (FGF‐4), insulin‐like growth factor 1 (IGF‐1), hepatocyte growth factor (HGF), platelet‐derived growth factor AB (PDGF‐AB), beta nerve growth factor (β‐NGF), tumor necrosis factor alpha (TNF‐α), interferon gamma (IFN‐γ), and interleukins (IL‐1 and IL‐6).

Given the breath of ADMSCs secretome constituents, these bioactive substances may counteract the adverse effects of corticosteroids. EGF, FGF‐4, and IGF‐1 promote keratinocyte and fibroblast proliferation via mitogen‐activated protein kinase/extracellular signal‐regulated kinase (MAPK/ERK) and phosphatidylinositol‐3 kinase/protein kinase B (PI3K/AKT) signaling pathways, thereby enhancing collagen and extracellular matrix production and restoring dermal thickness [11, 12]. HGF and PDGF‐AB further support fibroblast activation, angiogenesis, and tissue remodeling [13]. In addition, FGF and related paracrine signals from keratinocytes and fibroblasts have been shown to regulate melanocyte survival and melanogenesis, potentially facilitating repigmentation in corticosteroid‐damaged skin [14]. Collectively, these mechanisms may explain the gradual recovery of both pigmentation and skin contour observed in our patient (Figure 2).

**Figure FIGURE 2:**
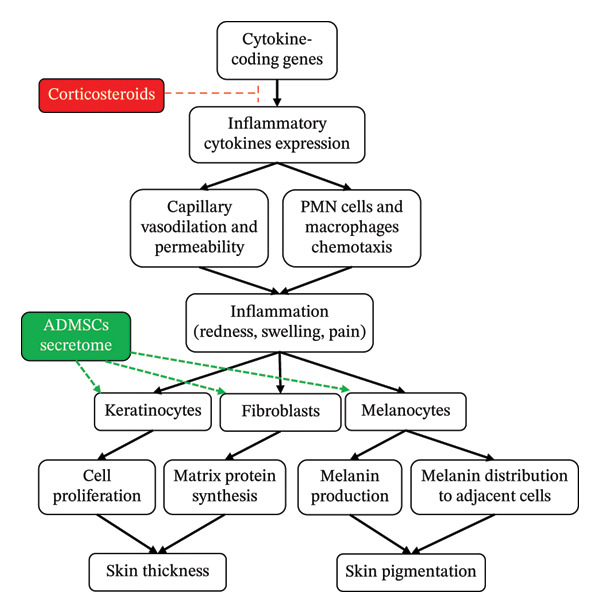
Schematic mechanisms of corticosteroids and ADMSCs secretome in the regulation of skin thickness and pigmentation [2, 3, 11–14]. The arrows and T bar represent activation and inhibition, respectively.

At first (Week 0), the ADMSCs secretome was administered through intradermal injection, but then we resorted to topical application following microneedling treatment in the subsequent clinical encounters (Weeks 2, 4, 6, and 8). The underlying reason was to minimize the pain experienced by the patient. Besides intradermal injection, microneedling may be an alternative method for topical drug administration. It establishes microtunnels with a depth of approximately 150 μm from the surface to allow transdermal ADMSCs secretome constituents delivery to the dermal layer [15]. Mechanical stimulation by microneedle also facilitates vascularization and collagen remodeling, possibly augmenting the effect of ADMSCs secretome to the treated skin [16].

Beyond post‐steroid skin changes, ADMSCs and their secretome have been increasingly explored in various dermatologic conditions, including photoaging, alopecia, chronic wounds, psoriasis, atopic dermatitis, and pigmentary disorders such as vitiligo. These applications leverage the paracrine effects of ADMSCs, which promote tissue regeneration, immunomodulation, and angiogenesis, while minimizing the risks associated with live cell transplantation [17]. Preclinical studies have demonstrated that mesenchymal stromal stem cell secretome accelerates wound closure, modulates inflammatory responses, promotes reepithelization, and enhances neovascularization in skin injury models [18]. Additionally, ADMSCs secretome induces fibroblasts proliferation, differentiation, and migration and also upregulates collagen Type I and Type III expressions, contributing to extracellular matrix remodeling and wound healing [19]. The present case adds to this growing body of evidence by suggesting a potential role for ADMSCs secretome in managing corticosteroid‐induced hypopigmentation and skin atrophy, particularly in elderly patients with limited skin regenerative capacity.

In conclusion, ADMSCs secretome may represent a favorable therapeutic candidate for postinflammatory hypopigmentation and skin atrophy induced by corticosteroids. Nevertheless, some limitations of this case report must be acknowledged. First, while pigmentation change and skin atrophy may be clinically evaluated by direct visual observation and comparing the lesion level to the adjacent skin through palpation, no objective measurements (e.g., histopathology or dermoscopy) were performed to quantify changes in pigmentation or skin thickness. Second, the natural tendency for post‐steroid changes to improve over time limits interpretation of the outcome, but the lack of clinical improvements in 5 months following the abstinence of corticosteroid injection suggested impaired recovery in our patient. Third, microneedling was performed as part of the regimen and may have also contributed to the clinical improvement in addition to the ADMSCs secretome. Finally, conclusions regarding the effectiveness of ADMSCs secretome cannot be drawn from a single uncontrollable observation. Hence, controlled studies involving several subjects are warranted to validate these findings and uncover the optimal dosage, administration method, duration of application, and long‐term safety before its implementation in clinical settings.

## Funding

No funding was received for this manuscript.

## Consent

The patient described in this case report has provided written informed consent for her details and photographs to be published.

## Conflicts of Interest

The authors declare no conflicts of interest.

## Data Availability

The data that support the findings of this study are available on request from the corresponding author. The data are not publicly available due to privacy or ethical restrictions.
